# Efficacy and safety of azvudine in patients with COVID-19: A systematic review and meta-analysis

**DOI:** 10.1016/j.heliyon.2023.e20153

**Published:** 2023-09-14

**Authors:** Zhaoyan Chen, Fangyuan Tian

**Affiliations:** a Department of Pharmacy, West China Hospital, Sichuan University, Chengdu, China; b Department of Epidemiology and Health Statistics, West China School of Public Health and West China Fourth Hospital, Sichuan University, Chengdu, China

**Keywords:** COVID-19, Azvudine, Randomized controlled trials, SARS-CoV-2, Meta-analysis

## Abstract

**Introduction:**

Azivudine has undergone a few randomized controlled trials (RCTs) as of late. This study aimed to assess the COVID-19 treatment with azvudine's efficacy and safety.

**Methods:**

Through January 20, 2023, systematic searches of PubMed, Embase, ClinicalTrials.gov, International Clinical Trials Registry Platform (ICTRP), Cochrane Central Register of Controlled Trials (CENTRAL), and MedRxiv were conducted to find the RCTs. The included studies' bias risk was evaluated using the Cochrane Handbook for Systematic Reviews of Interventions. Meta-analysis was performed using Revman 5.4 (PROSPERO Code: CRD42023395022).

**Results:**

A total of five RCTs with 1142 COVID-19 patients, 575 of whom received azvudine, were included. Additionally, seven RCTs are currently being conducted. In terms of clinical improvement and PT-PCR (reverse transcription polymerase chain reaction) negativity, the azvudine group had a greater patient percentage than the usual treatment or placebo group. It also took less time for the PT-PCR to become negative. In comparison to the placebo or standard treatment groups, the frequency of adverse events was reduced in the azvudine group (risk ratio [RR] = 0.89, 95% confidence interval [CI]: 0.80 to 0.99) and major adverse events (RR = 0.63, 95% CI: 0.22 to 1.79) groups.

**Conclusions:**

Without the burden of side effects, azvudine can hasten the clinical symptoms of COVID-19 patients and PT-PCR negative. It will take more extensive research to confirm these conclusions.

## 1 Introduction

Since December 2019, the coronavirus disease 2019 (COVID-19) epidemic has been reported in various parts of the world. To this day, COVID-19 continues to spread globally and pose a threat to human health [1,2]. As of February 2023, the World Health Organisation reported 753.4 million confirmed cases of COVID-19 worldwide, including 6.8 million fatalities [3]. So far, more than 1000 variant strains of the SARS-CoV-2 have been identified, and as the virus continues to mutate, Omicron has replaced Delta as the dominant variant, including BA.1, BA.2, BA.3, BA.4, BA.5 and their progeny lineages. Compared to the Delta variant, the Omicron variant is more infectious, less virulent, and has greater immune escape ability [4]. Compared with some previous COVID-19 viruses, once there is an infectious source, even if the COVID-19 vaccine is inoculated, the risk of infection is still increasing [5].

One year after contracting COVID-19, only 29% of patients had totally recovered, and 71% had sequelae, according to UK research on more than 2000 COVID-19 inpatients. Fatigue, muscle soreness, bodily slowness, poor sleep, and dyspnea are the most frequent side effects [6]. According to a study on Chinese patients, sequelae such weariness, muscle weakness, dyspnea, and sleep issues still plagued 55% of inpatients two years after contracting COVID-19. The health status of COVID-19 patients was noticeably worse two years later than the overall population [7]. The risk of organ failure and mortality is greatly increased with each COVID-19 infection, according to certain research, and increases over time. These conditions include those that impact the kidneys, diabetes, the lungs, heart, brain, blood, musculoskeletal system, and gastrointestinal tract, as well as mortality [8,9]. As a result, it's crucial to develop an efficient treatment strategy for COVID-19 patients [10] and to start using antiviral medications as soon as possible to treat COVID-19-related symptoms. Doing so can help patients' COVID-19 sequelae and lower their risk of hospitalization and mortality [11].

Remdesivir, chloroquine phosphate, and favipiravir are a few examples of broad-spectrum antiviral medications and medications targeting other particular viruses that were effective in combating the outbreak early on [12]. The research and development of oral small molecule medicines for SARS-CoV-2 have advanced significantly due to our growing understanding of the COVID-19 epidemic and SARS-CoV-2, and this has had a significant impact on the prevention and management of the epidemic [13]. Currently, RNA-dependent RNA polymerase (RdRp) and 3CLpro (3C-like protease) are being researched and developed as oral small molecule medicines for COVID-19 [14]. Azfudine, an oral antiviral drug independently developed in China, is a broad-spectrum RNA virus inhibitor that can inhibit recombinant HIV reverse transcriptase activity and can be combined with other drugs to treat AIDS (Acquired Immune Deficiency Syndrome). In addition, azivudine, as a nucleoside analogue of a synthetic viral RNA-dependent RNA polymerase (RdRp), is metabolized intracellular into an active 5 ′-triphosphate metabolite. This activator can act specifically on RdRp, embedding viral RNA during SARS-CoV-2 RNA synthesis. Thus, inhibiting SARS-CoV-2 replication, to achieve the role of COVID-19 treatment [15].

The National Medical Products Administration (NMPA) of China has currently cleared azivudine for emergency evaluation for common COVID-19. The published results do differ in a few ways, though. Meta-analyses on the effectiveness and safety of azvudine treatment for COVID-19 are yet lacking. In order to give more pertinent evidence for future therapeutic application, the goal of this study is to summarise and analyse the efficacy and safety of azvudine treatment for COVID-19.

## 2 Methods

### 2.1 Eligibility criteria

Studies were considered for inclusion if they satisfied the following requirements: (1) they involved COVID-19 patients; (2) they used azvudine treatment as the experimental drug; (3) they used placebo or standard treatment as controls, standard treatment were standard antiviral treatment and symptomatic treatment; (4) they were randomized controlled trials (RCTs); single-arm clinical trials were excluded from the study; and (5) they were unpublished RCTs with results available on preprints. Studies conducted in vitro, abstracts from posters or conferences, and studies lacking adequate data for outcome analysis were disregarded.

### 2.2 Search strategy and literature screening

The research was obtained from PubMed, Embase, ClinicalTrials.gov, International Clinical Trials Registry Platform (ICTRP), and MedRxiv from the time of publication until January 20, 2023. The search phrases “COVID-19,” “SARS-CoV-2,” and “azvudine” were used. Two investigators independently planned, carried out, and cross-checked all search tactics. The RCTs that were a part of our investigation were split into two groups. RCTs that have been published or that have not yet been published but have research findings fall into the first category, and RCTs that are actively being conducted fall into the second. To avoid overlooking any possibly pertinent studies, reference lists of selected papers were also looked through. The two researchers read the title and abstract of these articles back-to-back for preliminary screening after using EndNote X7 to filter duplicates. They then examined the full text of any articles that initially satisfied the inclusion criteria to decide whether they were ultimately included. The third researcher oversaw reaching an agreement when the other two researchers couldn't. The protocol (PROSPERO Code No. CRD42023395022) was submitted to the International Prospective Register of Systematic Reviews.

### 2.3 Data extraction

Each study that was included provided the following information, which was divided into three parts: (1) the fundamental details of the studies that were included, such as the initial author, publication year, clinical trial registration number, clinical trial phase, center, study population, group details, age range, and sample size; (2) PT-PCR (real-time polymerase chain reaction) negativity, adverse events, and serious adverse events are examples of outcomes to measure the effectiveness and safety of azivudine. (3) The quality of the included studies was evaluated using the Cochrane Collaboration's tool for evaluating risk of bias [16].

### 2.4 Statistical analysis

Based on the software Review Manager 5.4, a meta-analysis was carried out. The risk ratio (RR) and 95% confidence intervals (CI) were used to compute the outcomes. The heterogeneity between studies was examined using the Q statistic test and the I2 test. No statistical heterogeneity was deemed to exist if P > 0.05 and I2 50% were present. The fixed effects model was applied if there was no statistical heterogeneity between studies; otherwise, the random effects model was applied.

## 3 Results

### 3.1 Study selection

**Fig. 1:**
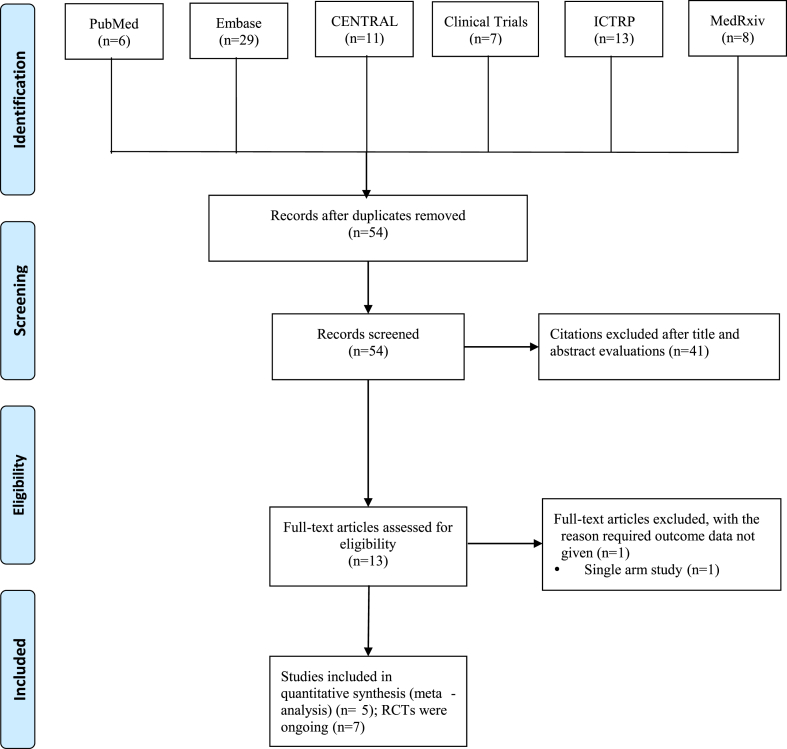
Flow chart of study selection.

A total of 74 papers were found through the search of pertinent databases, including 6 from PubMed, 29 from EMbase, 11 from CENTRAL, 20 active clinical trials from ClinicalTrials.gov and ICTRP, and 8 from the preprint system of articles MedRxiv. The titles and abstracts of 54 studies were evaluated, and 41 papers were eliminated after 20 duplicate studies were eliminated. 13 studies were additionally eliminated after reading the complete texts. The meta-analysis includes 5 studies [[17], [18], [19], [20]] in total (Fig. 1; Table 1). Additionally, the information from 7 ongoing trials was gathered (Fig. 1; Table 2).

**Table 1:** Characteristics of the included published studies.

Article	Clinical trials registration	Phase	Center	Study population	Intervention group	Control group	Age range	Sample size	
								Intervention group	Control group
Cabral et al., 2022a [17]	NCT04668235	3	Single-center	Patients with moderate COVID-19	Azvudine 5 mg + standard treatment	Placebo + standard treatment	18 Years and older	89	83
Cabral et al., 2022b [18]	NCT05033145	3	Single-center	Patients with mild COVID-19	Azvudine 5 mg + standard treatment	Placebo + standard treatment	18 Years and older	143	138
Ren et al., 2020 [19]	ChiCTR2000029853	3	Single-center	Patients with mild and common COVID-19	Azvudine 5 mg + standard treatment	Placebo + standard treatment	18 Years and older	10	10
China, 2022 [20]	NCT04746183	3	Multi-center	Patients with mild and common COVID-19	Azvudine 5 mg + standard treatment	Placebo + standard treatment	18 Years and older	174	174
Russia, 2022 [20]	NR	NR	Multi-center	Patients with moderate COVID-19	Azvudine 5 mg	Placebo	18 Years and older	157	157

NR: Not reported.

**Table 2:** Characteristics of included ongoing studies.

Clinical trials registration	Status	phase	Center	Location	Intervention	Control	Age range	Sample size
ChiCTR2200067174	Recruiting	Phase 4	Single-center	China	Azvudine + Traditional Chinese medicine	Traditional Chinese medicine	aged 18–65 years	100
ChiCTR2100052875	Recruiting	Phase 3	Single-center	China	Azvudine + standard treatment	Standard treatment	aged 18–60 years	60
NCT05689034	Not Yet Recruiting	Phase 2-3	Multi-center	China	Azvudine	Placebo	18 years and older	1096
NCT05682599	Recruiting	Phase 2	Single-center	China	Azvudine	Placebo	aged 18–65 years	300
NCT05633433	Recruiting	Phase 2-3	Multi-center	China	Azvudine	Placebo	18 years and older	1550
NCT05697055	Not Yet Recruiting	Phase 4	Multi-center	China	Azvudine	Paxlovid	18 years and older	410
NCT05642910	Recruiting	NR	Single-center	Malaysia	Azvudine	Paxlovid	aged 18–85 years	540

NR: Not reported.

### 3.2 Study characteristics

1142 participants from a total of five RCTs were included in the study. 575 of these patients were assigned to azvudine treatment plans. On the clinical trial registration website, the protocols for four trials have been made public, including two multicenter investigations. The majority of the study's participants were patients with mild or moderate COVID-19. In the intervention group, patients received either 5 mg of azvudine or azvudine in addition to normal care. Table 3 displays the caliber of the included studies. The study's overall caliber is not very high. Azvudine is now being used in around 7 RCTs that have been registered with ICTRP or ClinicalTrials.gov. Table 2 displays specifics of various RCTs.

**Table 3:** Quality assessment of included studies.

study	Selection bias		Performance bias	Detection bias	Attrition bias	Reporting bias	Other bias	Total score (max = 7)
	Random sequence generation	Allocation concealment	Blinding of participants and personnel	Blinding of outcome assessment	Incomplete outcome data	Selective reporting		
Cabral et al., 2022a [17]	+	+	+	+	+	?	?	5
Cabral et al., 2022b	+	+	+	+	+	?	?	5
Cabral et al., 2022b [18]	+	+	+	+	+	+	+	7
Ren et al., 2020 [19]	?	?	+	+	?	?	?	2
Russia, 2022	?	?	+	+	?	?	?	2

### 3.3 Efficacy of azvudine

The results of the study conducted in Russia showed that the proportion of subjects with clinical improvement was significantly higher than that of the control group on the 7th day after the first administration of azvudine (Azvudine group: 57/157, control group: 15/157, P < 0.001), and the median time of clinical improvement was significantly shorter (Azvudine group: 10 days, control group: 13 days, P < 0.001). The results of the study conducted in China showed that there was no statistically significant difference in the changes of viral load between the azvudine group and the control group. The results of another single-center study conducted in China showed that the proportion of RT-PCR negativity in azivudine group was higher than that in the control group, and the median time of RT-PCR negativity was significantly shorter (Azvudine group: 2.6 days, control group: 5.6 days, P = 0.008). The results of the study on the treatment of moderate COVID-19 patients in Brazil showed that the first RT-PCR negativity (Azvudine group: 6.24 days, control group: 7.94 days, P = 0.002) and hospital stay (Azvudine group: 6.5 days, control group: 7.73 days, P = 0.028) in the Azvudine group were shorter than those in the control group. The results of the study on the treatment of mild COVID-19 patients in Brazil showed that the first RT-PCR negativity (Azvudine group: 5.55 days, control group: 8.27 days, P < 0.001) in the Azvudine group was shorter than those in the control group.

### 3.4 Safety of azvudine

**Fig. 2:**
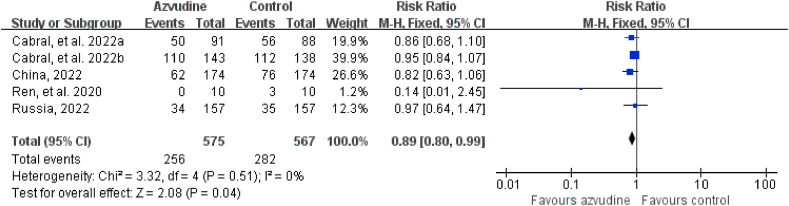
Forest plot of the comparison of adverse events between azvudine treatment and control.

**Fig. 3:**
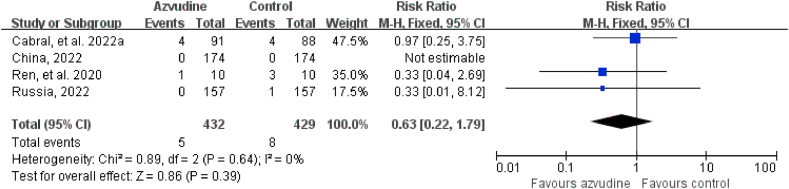
Forest plot of the comparison of serious adverse events between azvudine treatment and control.

Five RCTs reported adverse events and serious adverse events. The incidence of adverse events in COVID-19 patients in the intervention groups who received azvudine of treatment was 44.52% (256/575) and in the control groups 49.74% (282/567), respectively. The incidence of serious adverse events in COVID-19 patients in the intervention groups who received azvudine of treatment was 1.16% (5/432) and in the control groups 1.86% (8/429), respectively. The safety of azvudine was better than that in the control group (adverse events: RR = 0.89, 95% CI: 0.80 to 0.99, P = 0.04; serious adverse events: RR = 0.63, 95% CI: 0.22 to 1.79, P = 0.39) (Fig. 2, Fig. 3). The subgroup analysis of the safety of included published studies were showed in Table 4.

**Table 4:** The subgroup analysis of included published studies.

Analysis	No. of studies	Sample size	OR	95% CI	*P*
Brazil					
Adverse events	2	460	0.92	0.82 to 1.03	0.15
Serious adverse event	1	179	0.97	0.25 to 3.75	0.96
China					
Adverse events	2	368	0.79	0.61 to 1.02	0.07
Serious adverse event	2	368	0.33	0.04 to 2.69	0.3
Russia					
Adverse events	1	314	0.97	0.64 to 1.47	0.89
Serious adverse event	1	314	0.33	0.01 to 8.12	0.5

## 4 Discussion

For the treatment of common COVID-19 patients with advanced severe high-risk factors, such as advanced age, not having received the COVID-19 vaccine, chronic kidney disease, diabetes, severe cardiovascular disease, chronic obstructive pulmonary disease, organ transplant recipients, and other individuals taking immunosuppressive medications, azvudine has been approved for import registration. On July 25, 2022, it received its formal launch, following paxlovid as the second small molecule oral medication for COVID-19 in mainland China. On January 6, 2023, the People's Republic of China's National Health Commission published the Scheme for Diagnosis and Treatment of SARS-CoV-2 (The 10th Trial Edition). Compared with the 9th Edition of the diagnosis and treatment plan previously released, in addition to paxlovid, monoclonal antibody, intravenous injection of human immunoglobulin and convalescent plasma, the antiviral treatment content added azvudine.

Efficacy is an important indicator of azvudine treatment for COVID-19. Azvudine can significantly increase the proportion of patients with improved clinical symptoms of COVID-19 within 5 days of treatment and achieve excellent clinical results. In terms of inhibiting COVID-19, it can inhibit the activity of COVID-19 and increase the proportion of PT-PCR negativity. Therefore, it is better to use azvudine in the early stage of infection with SARS-CoV-2. This is mainly because azvudine acts through RdRp to induce the production of the ARS-COV-2 wrong mutation, thus inhibiting the replication of ARS-COV-2. At the initial stage of infection, the virus replicates a lot, so the application of azvudine has the best effect during this period. Therefore, the window period of most antiviral drugs is only five days after the symptoms first appear, and the use of such drugs as the disease progresses is not effective. A clinical study found that only half of COVID-19 infections can be detected within five days after symptoms appear [21]. Such a short window has become a major defect in the use of such drugs. Our study found that patients who received azvudine within 7–15 days still had better clinical improvement. The results showed that the time of RT-PCR negativity was significantly shorter, this is similar to the results of another study conducted in China [22]. However, the morality and hospitalization rate of patients with COVID-19 receiving azvudine treatment has not been reported in these RCTs, and many statistical information disclosures is not detailed enough. Limited to this, it is difficult for us to conduct a meta-analysis of the efficacy indicators of azvudine. Further real-world studies are needed to confirm whether azvudine has better long-term clinical effects than other drugs.

In terms of safety, azvudine had a good performance compared with the control group in terms of adverse events or series adverse events, which indicates that azvudine has good safety. The most common adverse effects of azvudine were fever, headache, dizziness, nausea, vomiting and diarrhea, with the severity of grade 1 to 2. However, most of the clinical trials have not yet published their formal research results, and the current research sample size is small, this conclusion still needs further exploration in follow-up research. Azivudine is a P-glycoprotein (P-gp) substrate and a weak P-gp inducer [23,24]. Based on this, it is speculated that the combination of P-gp substrates (such as digoxin, dabigatran, colchicine) and P-gp inhibitors (such as cyclosporin, itraconazole, voriconazole, ritonavir, dronedarone, amiodarone, verapamil, clarithromycin, grapefruit juice) and P-gp inducers (such as rifampin, St. John's wort extract) should be careful. If combined use is necessary, blood drug concentration monitoring shall be carried out when necessary.

The main advantage of this study is that it is the first fast review and meta-analysis on the effectiveness and safety of azvudine treatment in patients with COVID-19 to the best of our knowledge. To lessen any bias, this article underwent thorough quality evaluation procedures. For a thorough assessment of the safety of azvudine treatment for COVID-19, strict and practical inclusion criteria were also used in this meta-analysis, and only randomized clinical studies were included. This study does, however, have certain shortcomings. First, the number of research that may be added from the real world is still quite small. There is a significant disparity in the sample size, outcome indicators, and population of RCTs in the study, and there are relatively few data about the effectiveness of azvudine on COVID-19 patients in clinical practice. Additionally, this article's poor quality prevented it from performing a subgroup analysis based on risk factors including older age and vaccination status, which could have impacted the accuracy of the results. Therefore, more extensive and high-quality studies are still needed to confirm the effectiveness and safety of azvudine treatment for COVID-19.

## Author contribution statement

All authors listed have significantly contributed to the development and the writing of this article.

## Data availability statement

Data will be made available on request.

## Ethics statement

Not required.

## Funding

This work was supported by the Sichuan Science and Technology Program (Project number: 2022JDR0326, 2023JDR0246).

## Declaration of competing interest

The authors declare that they have no known competing financial interests or personal relationships that could have appeared to influence the work reported in this paper.
